# Depletion of highly abundant proteins from human cerebrospinal fluid: a cautionary note

**DOI:** 10.1186/s13024-015-0050-7

**Published:** 2015-10-15

**Authors:** Ramona Günther, Eberhard Krause, Michael Schümann, Ingolf E. Blasig, Reiner F. Haseloff

**Affiliations:** Leibniz Institute of Molecular Pharmacology, Robert-Roessle-Str. 10, D-13125 Berlin, Germany

**Keywords:** Biomarker, Human cerebrospinal fluid, Albumin depletion, Unspecific binding, Quantitation, Mass spectrometry

## Abstract

Affinity-based techniques, both for enrichment or depletion of proteins of interest, suffer from unwanted interactions between the bait or matrix material and molecules different from the original target. This effect was quantitatively studied by applying two common procedures for the depletion of albumin/gamma immunoglobulin to human cerebrospinal fluid. Proteins of the depleted and the column-bound fraction were identified by mass spectrometry, employing ^18^O labeling for quantitation of their abundance. To different extents, the depletion procedures caused the loss of proteins previously suggested as biomarker candidates for neurological diseases. This is an important phenomenon to consider when quantifying protein levels in biological fluids.

**Electronic supplementary material:**

The online version of this article (doi:10.1186/s13024-015-0050-7) contains supplementary material, which is available to authorized users.

## Background

Affinity chromatography-based enrichment or depletion techniques are of great importance in both basic and applied protein research in the biomedical field. Many different materials are utilized for binding specific targets - ranging from native (e.g., immunoglobulins), or tagged proteins/protein domains to smaller structures such as synthetic peptides. Protein-protein interaction studies in or protein purification from complex environments are unthinkable without co-immunoprecipitation protocols or other types of pull-down assays. On the other hand, the search for biomarkers using proteomic methods can be facilitated after depletion of highly abundant proteins from biological fluids [1]. However, affinity-based techniques suffer from an annoying disadvantage: non-specific binding, either to the bait molecule or to the matrix material, can significantly impair the quality of the experiment. False positive results may arise or potential biomarkers can be removed from the biological sample.

Human cerebrospinal fluid (hCSF) experiences increasing interest as a source of biomarkers of neurological diseases [2]. In the present contribution, two common principles of albumin and immunoglobulin removal, Cibacron Blue/Protein A (CB-D)- and antibody/Protein G-based (AB-D) depletion, have been tested with respect to their specificity when applied to hCSF. Although the problem is qualitatively described in the literature, quantitative data on non-specific binding occurring in affinity approaches (which are important, e.g., for the reliable identification of potential biomarkers) are not available so far. Here, we use mass spectrometry (MS)-based protein identification combined with stable isotope labeling by incorporation of ^18^O for relative quantification of co-depleted proteins [3]. The results demonstrate that the abundance of numerous proteins, including many biomarker candidates, is strongly influenced by depletion procedures.

### Co-depletion removes potential biomarker proteins

The depletion of albumin and immunoglobulins was accomplished by application of two different approaches, CB-D and AB-D (Additional file 1: for experimental details). Briefly, the column-bound and depleted fractions were collected and separated by one-dimensional sodium dodecyl sulfate gel electrophoresis. In-gel digestion of both lanes using trypsin was performed for the column-bound fraction in H_2_^18^O and for the flow-through fraction in normal water. Peptide extracts originating from gel slices of identical molecular weight were combined. Subsequent mass spectrometry identified the proteins and their respective depletion ratios R = I_c_/I_d_ (mass spectra intensities of column-bound vs. depleted fraction) via analysis of the isotope distribution.

**Fig. 1:**
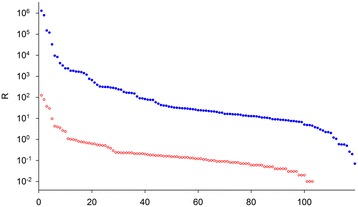
Distribution of ratios R (R = I_c_/I_d_, I_c_, I_d_, mass spectrometry signal intensities of proteins [mean of corresponding peptide ratios] in column-bound/depleted fractions) for Cibacron Blue/Protein A- (blue filled circles) and antibody/Protein G-based depletion (red open circles)

The Coomassie-stained gels (Additional file 2: Figure S1) demonstrate that both depletion procedures used for the experiments removed albumin and IgGs from the hCSF sample. The efficacy of albumin depletion was determined by densitometric analysis of the main albumin gel bands (I_column-bound_/I_depleted_ = 0.59 for CB-D, 2.41 for AB-D). The gel bands of the column-bound fraction indicate that there is considerable co-depletion of proteins, in particular after application of CB-D. Preliminary experiments directed at analyzing the identities of proteins in the column-bound fractions revealed overwhelming dominance of albumin fragments in gel bands with apparent molecular masses ≤64 kDa. Thus, MS-based quantitative evaluation was carried out for gel slices covering all proteins with apparent molecular masses above the albumin band. An overview of the vulnerability of both procedures for co-depletion is shown in Fig. 1, which presents the distribution of the incidence of depletion ratios R.

For the CB-D method, 17 of the entries with R ≥ 50 refer to immunoglobulins (24 entries in total, Additional file 3: Table S1) identified with ratios indicating almost complete elimination from the sample. However, there is also efficient co-depletion: 28 proteins different from immunoglobulins are found at more than 50-fold excess in the column-bound fraction also indicating virtually total loss in the depleted fraction. These 28 gene products include 24 proteins (selection given in Table 1) which have been previously classified as potential biomarkers for specific (preferentially neurodegenerative) diseases. The candidate marker proteins with the highest depletion-caused loss include junction plakoglobin (suggested as a marker of atherosclerosis [4]), colony-stimulating factor 1 receptor (marker candidate of amyotrophic lateral sclerosis [5]) and plasminogen (marker candidate of Alzheimer’s disease (AD) [6]). Differential expression has been demonstrated for complement C5, ectonucleotide pyrophosphatase/phosphodiesterase family member 2 and α-2-macroglobulin in the CSF of CNS lymphoma patients as well as for complement C7 and coagulation factor V in choroid plexus tumors [7].

**Table 1:** Proteins identified in column-binding fractions (selection)

Protein	Accession	R	Reference
Cibacron Blue/Protein A – based depletion			
Junction plakoglobin	sp|P14923	>50	[4]
Complement component C7	sp|P10643	>50	[7]
Complement C5	sp|P01031	>50	[7]
Plasminogen	sp|P00747	>50	[8]
Colony-stimulating factor 1 receptor	tr|E9PEK4	>50	[5]
Ectonucleotide pyrophosphatase/phosphodiesterase 2	tr|E7EUF1	>50	[7]
Alpha-2-macroglobulin	sp|P01023	>50	[6]
Coagulation factor V	sp|P12259	>50	[7]
Complement factor B	tr|B4E1Z4	>50	[6]
Complement C1r subcomponent	sp|P00736	>50	[7]
Gelsolin	sp|P06396	>50	[6]
Isoform 2 of amyloid-like protein 1	sp|P51693-2	>50	[6]
Fibulin-1	sp|P23142	>50	[7]
Complement C2	sp|P06681	>50	[6]
Complement factor H	sp|P08603	>50	[9]
Neurexin-2-alpha	sp|Q9P2S2	>50	[8]
Complement C3	sp|P01024	>50	[8]
Antibody/Protein G – based depletion			
Desmoglein-1	sp|Q02413	>50	[8]
Calmodulin-like protein 5	sp|Q9NZT1	50 > R > 20	[8]
Collagen alpha-1(I) chain	sp|P02452	20 > R > 2	[8]
Collagen, alpha-2(I) chain	tr|F5H299	20 > R > 2	[6]
Complement factor H	sp|P08603	2 > R > 0.5	[9]
Plasminogen	sp|P00747	2 > R > 0.5	[8]
Alpha-1-antitrypsin	sp|P01009	2 > R > 0.5	[9]
Isoform 2 of calsyntenin-1	sp|O94985-2	2 > R > 0.5	[9]

Accession, accession number in SwissProt (sp)/Tremble (tr) data base; R, MS signal intensity ratio I_column-bound_/I_depleted_; Ref., reference suggesting eligibility as a biomarker; complete lists of identified proteins available as additional files (Additional files 3 and 4: Tables S1 and S2)

Much lower protein loss due to co-depletion was observed after antibody-based depletion (Table 1, complete results in Additional file 4: Table S2). Nevertheless, several potential biomarker proteins were also found to dominate the column-bound fractions. Desmoglein-1, calmodulin-like protein 5, collagen alpha-1(I) chain and plasminogen have been identified as marker candidates for multiple sclerosis [8] while increased levels of α-1-antitrypsin and calsyntenin-1 (isoform 2) have been found in the CSF of AD and Parkinson’s disease patients, respectively [9].

### Depletion protocols: caution advised

Non-specific binding is inherent in affinity-based enrichment or depletion protocols. The efficacy of the Cibracon Blue/Protein A-based procedure for immunoglobulin depletion was found significantly higher as compared to the antibody-based method which in turn showed a lower co-depletion. For albumin and immunoglobulins, non-specific association overlaps with the functional binding of these proteins to their target molecules present in biological fluids or cells. Recent data obtained using identical depletion techniques for canine CSF indicate that indeed both phenomena occur [10]. However, for the practical problem of the depletion of highly abundant proteins, it is obviously irrelevant which mechanisms (non-specific binding to matrix/bait or specific binding to bait molecule) cause the observed co-depletion.

## Conclusion

For affinity approaches, there is a lack of quantitative data on unwanted removal (or enrichment) of proteins, although this information is of crucial importance for assessing the quality of an experiment and the reliability of its results. With respect to depletion procedures, our quantitative approach demonstrated that many proteins previously identified as potential biomarkers are completely removed from the hCSF sample, often with even higher efficiency than the original target of the procedure. The supplemental tables give (non-exhaustive) lists of proteins that were particularly affected in these experiments. Taking this into account, it is obvious that the quantification of the abundance of many proteins is prone to major systematic errors when the sample preparation includes depletion protocols of the types investigated here. Moreover, the presented data, although obtained for depletion procedures, can also be relevant for approaches based on similar protocols for affinity enrichment.

## Additional files

Additional file 1:
**Detailed description of sample preparation and experimental procedures.** (PDF 102 kb) Additional file 2: Figure S1. Protein fractions obtained by depletion of human cerebrospinal fluid (CSF), separated by sodium dodecyl sulfate gel electrophoresis and stained by Coomassie Brilliant Blue G250. (Protein marker, M; CSF undepleted, C; depleted (=flow-through) fraction, FT; column-bound (=eluate) fraction, E); protein load, 5 μg/lane. (PDF 71 kb) Additional file 3: Table S1. Proteins identified in fractions obtained by Cibacron Blue/Protein A-based depletion (Accession, accession number in SwissProt/Tremble data base; R, MS signal intensity ratio I_column-bound_/I_depleted_; Score, Mascot Score; Reference, reference suggesting eligibility as a biomarker). (PDF 34 kb) Additional file 4: Table S2. Proteins identified in fractions obtained by antibody/Protein G-based depletion (Accession, accession number in SwissProt/Tremble data base; R, MS signal intensity ratio I_column-bound_/I_depleted_; Score, Mascot Score; Reference, reference suggesting eligibility as a biomarker). (PDF 27 kb)

## Abbreviations

hCSF: Human cerebrospinal fluid
CB-D: Cibacron Blue/Protein A-based depletion
AB-D: Antibody/Protein G-based depletion
MS: Mass spectrometry
AD: Alzheimer’s disease

## References

[1] Roche S, Tiers L, Provansal M, Seveno M, Piva MT, Jouin P (2009). Depletion of one, six, twelve or twenty major blood proteins before proteomic analysis: the more the better?. J Proteomics.

[2] Potter WZ (2012). Mining the secrets of the CSF: developing biomarkers of neurodegeneration. J Clin Invest.

[3] Lange S, Sylvester M, Schümann M, Freund C, Krause E (2010). Identification of phosphorylation-dependent interaction partners of the adapter protein ADAP using quantitative mass spectrometry: SILAC vs O-18-labeling. J Proteome Res.

[4] Cooksley-Decasper S, Reiser H, Thommen DS, Biedermann B, Neidhart M, Gawinecka J (2012). Antibody phage display assisted identification of junction plakoglobin as a potential biomarker for atherosclerosis. PLoS One.

[5] Tanaka M, Kikuchi H, Ishizu T, Minohara M, Osoegawa M, Motornura K (2006). Intrathecal upregulation of granulocyte colony stimulating factor and its neuroprotective actions on motor neurons in amyotrophic lateral sclerosis. J Neuropath Exp Neur.

[6] Kroksveen AC, Opsahl JA, Aye TT, Ulvik RJ, Berven FS (2011). Proteomics of human cerebrospinal fluid: discovery and verification of biomarker candidates in neurodegenerative diseases using quantitative proteomics. J Proteomics.

[7] Hasselblatt M, Bohm C, Tatenhorst L, Dinh V, Newrzella D, Keyvani K (2006). Identification of novel diagnostic markers for choroid plexus tumors - a microarray-based approach. Am J Surg Pathol.

[8] Noben JP, Dumont D, Kwasnikowska N, Verhaert P, Somers V, Hupperts R (2006). Lumbar cerebrospinal fluid proteome in multiple sclerosis: characterization by ultrafiltration, liquid chromatography, and mass spectrometry. J Proteome Res.

[9] Yin GN, Lee HW, Cho JY, Suk K (2009). Neuronal pentraxin receptor in cerebrospinal fluid as a potential biomarker for neurodegenerative diseases. Brain Res.

[10] Günther R, Krause E, Schümann M, Ausseil J, Heard JM, Blasig IE (2014). Removal of albumin and immunoglobulins from canine cerebrospinal fluid using depletion kits: a feasibility study. Fluids Barriers CNS.

