# Supplementary material for: Depletion of highly abundant proteins from human cerebrospinal fluid: a cautionary note
Source: Mol Neurodegener. 2015 Oct 15;10:53. doi: 10.1186/s13024-015-0050-7 (PMC4608131; doi:10.1186/s13024-015-0050-7)
Supplement: Additional file 1: — Detailed description of sample preparation and experimental procedures. (PDF 102 kb) [file 13024_2015_50_MOESM1_ESM.pdf]

Title: Depletion of highly abundant proteins from human cerebrospinal fluid: a cautionary note

Journal: Molecular Neurodegeneration

Authors: Ramona Günther, Eberhard Krause, Michael Schümann, Ingolf E. Blasig, Reiner F. Haseloff

Corresponding author: Reiner F. Haseloff, Leibniz Institute of Molecular Pharmacology, Robert-Roessle-Str. 10, D-13125 Berlin, Germany; email, haseloff@fmp-berlin.de

Sample preparation and experimental procedures

Human CSF (hospital remnants) was obtained from Seralab (Sera Laboratories International, Haywards Heath/UK). Pooled CSF samples of two individual donors were centrifuged (2000×g, 10 min) to remove cells or other insoluble substances. As recommended for proteomic analyses [1], samples were pretreated by acetone precipitation, a procedure which increases albumin depletion from CSF without affecting unspecific binding [2].

For albumin and IgG depletion based on an affinity resin column, we used a kit (ProteoSeek™, Thermo-Fisher Scientific, Bonn/Germany) which utilizes a classical mixed bed of Cibracon Blue/Protein A and removes human serum albumin (HSA) and the major subclasses of γ-globulin from serum, plasma or spinal fluids. According to the manufacturer, this kit has given favorable results with human, monkey, swine and rabbit samples.

Antibody-based depletion of albumin and IgG was applied for comparison. The Vivapure Anti-HSA/IgG Kit (Sartorius, Göttingen/Germany) removes HSA and IgG from body fluids such as serum and plasma. Albumin depletion is accomplished with a unique antibody fragment coupled to a low-binding, cross-linked agarose, and IgG removal is achieved with Protein G ligand.

Depletion procedures for hCSF samples (100 µg protein each) were performed according to protocols provided by the kit manufacturers. The flow-through (*i.e.*, depleted) fractions of CSF were collected, elutions from the depletion columns were carried out by adding Laemmli

buffer (without reducing agents) to the columns. Cysteines were reduced by adding dithiothreitol (10 mM, 60°C, 15 min), followed by alkylation with iodoacetamide (55 mM, 21°C, 15 min) in the dark. All protein fractions were separated on Novex Tris/glycine 4-20% gels (Life Technologies, Darmstadt/Germany). Proteins were stained with Coomassie G-250 (Serva, Heidelberg/Germany). For both kits, three independent experiments were carried out, each with a pooled hCSF sample.

As described [3], complete gel lanes above the albumin band were cut in slices, and peptides were isotopically labeled by enzymatic digestion using trypsin (50 ng, sequencing grade modified, Promega) in the presence of $H_2{}^{18}O$ (Sigma Aldrich, 97% ^{18}O) and $H_2{}^{16}O$ for eluted proteins and proteins of the flow-through fraction, respectively. Samples from paired gel slices were combined immediately before MS analysis. Liquid chromatography tandem mass spectrometry (LC-MS/MS) analyses were performed on a LTQ-Orbitrap XL mass spectro-meter (Thermo Fisher) equipped with an Eksigent 2D nanoflow LC system (Axel Semrau GmbH, Sprockhövel/Germany). Mass spectra were acquired in a data-dependent mode. Automatic gain control was set to 1×10^6 and 20,000 for Orbitrap-MS and LTQ-MS/MS scans, respectively. Generated peak lists and the MASCOT server (version 2.2, Matrix Science Ltd., London/UK) were used to search against the SwissProt database (version 56.2). A maximum of two missed cleavages was allowed, mass tolerance of precursor and sequence ions was 10 ppm and 0.35 Da, respectively. The program Scaffold (v2.02, Proteome Software Inc.) was used to validate MS/MS-based peptide and protein identifications. Protein identifications were accepted if they could be established at greater than 99.0% probability and contained at least 2 identified peptides. Quantification was carried out using the Mascot Distiller Quantitation Toolbox (version 2.2.1.2, Matrix Science) and was based on calculations of $^{18}O/^{16}O$ isotope intensity ratios $R=I_{column\text{-}bound}/I_{depleted}=I_c/I_d$ of at least two identified peptides. Proteins were listed when identified in at least two independent experiments.

Reference List

1. Chen RL, Sage EA, Dunn MJ, Wait R, Preston JE. Optimising ovine cerebrospinal fluid preparation for two-dimensional gel electrophoresis**.** Proteomics. 2006;6:3170-5.

2. Günther R, Krause E, Schümann M, Ausseil J, Heard JM, Blasig IE *et al*. Removal of albumin and immunoglobulins from canine cerebrospinal fluid using depletion kits: a feasibility study**.** Fluids Barriers CNS. 2014;11:14.

3. Lange S, Sylvester M, Schümann M, Freund C, Krause E. Identification of Phosphorylation-Dependent Interaction Partners of the Adapter Protein ADAP using Quantitative Mass Spectrometry: SILAC vs O-18-Labeling**.** J Proteome Res. 2010;9:4113-22.
