# Supplementary material for: Depletion of highly abundant proteins from human cerebrospinal fluid: a cautionary note
Source: Mol Neurodegener. 2015 Oct 15;10:53. doi: 10.1186/s13024-015-0050-7 (PMC4608131; doi:10.1186/s13024-015-0050-7)
Supplement: Additional file 2: Figure S1. — Protein fractions obtained by depletion of human cerebrospinal fluid (CSF), separated by sodium dodecyl sulfate gel electrophoresis and stained by Coomassie Brilliant Blue G250. (Protein marker, M; CSF undepleted, C; depleted (=flow-through) fraction, FT; column-bound (=eluate) fraction, E); protein load, 5 μg/lane. (PDF 71 kb) [file 13024_2015_50_MOESM2_ESM.pdf]

Title: Depletion of highly abundant proteins from human cerebrospinal fluid: a cautionary note

Journal: Molecular Neurodegeneration

Authors: Ramona Günther, Eberhard Krause, Michael Schümann, Ingolf E. Blasig, Reiner F. Haseloff

Corresponding author: Reiner F. Haseloff, Leibniz Institute of Molecular Pharmacology, Robert-Roessle-Str. 10, D-13125 Berlin, Germany; email, haseloff@fmp-berlin.de

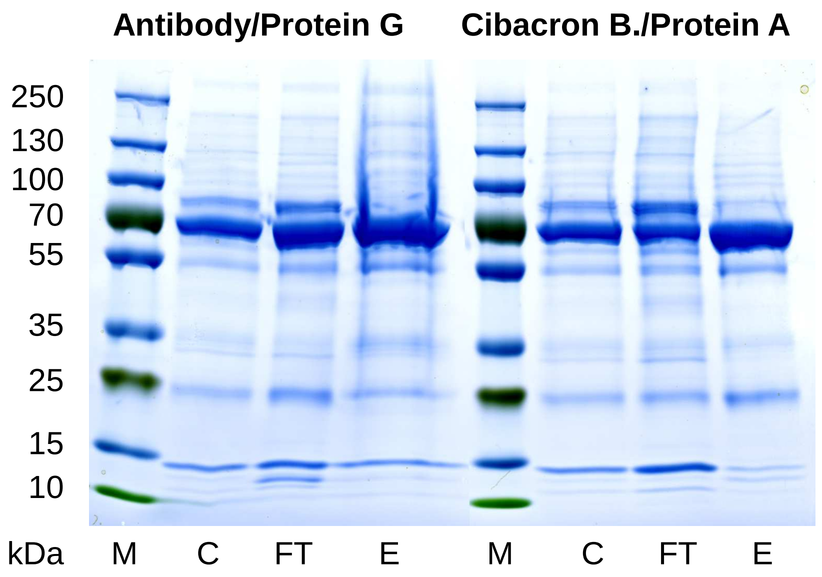

Supplementary Figure 1: Protein fractions obtained by depletion of human cerebrospinal fluid (CSF), separated by sodium dodecyl sulphate gel electrophoresis and stained by Coomassie Brilliant Blue G250. (Protein marker, M; CSF undepleted, C; depleted (=flow-through) fraction, FT; column-bound (=eluate) fraction, E); protein load, 5 µg/lane
