# Supplementary material for: Depletion of highly abundant proteins from human cerebrospinal fluid: a cautionary note
Source: Mol Neurodegener. 2015 Oct 15;10:53. doi: 10.1186/s13024-015-0050-7 (PMC4608131; doi:10.1186/s13024-015-0050-7)
Supplement: Additional file 3: Table S1. — Proteins identified in fractions obtained by Cibacron Blue/Protein A-based depletion (Accession, accession number in SwissProt/Tremble data base; R, MS signal intensity ratio Icolumn-bound/Idepleted; Score, Mascot Score; Reference, reference suggesting eligibility as a biomarker). (PDF 34 kb) [file 13024_2015_50_MOESM3_ESM.pdf]

Title: Depletion of highly abundant proteins from human cerebrospinal fluid: a cautionary note

Journal: Molecular Neurodegeneration

Authors: Ramona Günther, Eberhard Krause, Michael Schümann, Ingolf E. Blasig, Reiner F. Haseloff

Corresponding author: Reiner F. Haseloff, Leibniz Institute of Molecular Pharmacology, Robert-Roessle-Str. 10, D-13125 Berlin, Germany; email, haseloff@fmp-berlin.de

Supplementary Table 1: Proteins co-depleted in eluate/flow-through fractions obtained by Cibacron Blue/Protein A-based albumin/Ig depletion of human cerebrospinal fluid

**Protein**	**Accession**	**Score**	**Mass (Da)**	**R**	**Reference**
Ig lambda chain V-IV region Hil	sp|P01717	64	11510	1291360.45	
Junction plakoglobin	sp|P14923	225	81693	787005.00	[1]
Ig heavy chain V-III region GAL	sp|P01781	295	12722	149151.86	
Ig heavy chain V-III region VH26	sp|P01764	434	12574	119590.67	
Ig heavy chain V-III region BUT	sp|P01767	512	12371	32686.84	
Complement component C7	sp|P10643	1425	93457	9479.54	[2]
Vitamin K-dependent protein S	sp|P07225	535	75074	8261.41	
Ig heavy chain V-II region NEWM	sp|P01825	320	12782	4190.77	
Complement C5	sp|P01031	387	188186	3245.50	[2]
Plasminogen	sp|P00747	1173	90510	2374.78	[3]
Colony-stimulating factor 1 receptor	tr|E9PEK4	601	74222	2364.82	[4]
Ectonucleotide pyrophosphatase/phosphodiesterase 2	tr|E7EUF1	1573	101488	1824.75	[2]

Ig lambda-2 chain C regions	sp|P0CG05	985	11287	1822.29	
Immunoglobulin lambda-like polypeptide 5	sp|B9A064	797	23049	1675.48	
Ig kappa chain V-IV region Len	sp|P01625	235	12632	1637.05	
Interleukin-6 receptor subunit beta	sp|P40189	106	96216	1575.19	[5]
Alpha-2-macroglobulin	sp|P01023	8149	164837	1414.75	[2], [6]
Coagulation factor V	sp|P12259	152	251546	1193.43	[2]
Ig lambda chain V-III region LOI	sp|P80748	164	11928	758.40	
Complement factor B	tr|B4E1Z4	2063	140853	661.01	[6]
Complement C1r subcomponent	sp|P00736	985	80067	502.98	[2]
Gelsolin	sp|P06396	2367	85644	395.63	[6,7]
Ig gamma-4 chain C region	sp|P01861	1067	35918	327.77	
Receptor-type tyrosine-protein phosphatase S	sp|Q13332	323	217041	314.10	[8]
Ig gamma-3 chain C region	sp|P01860	1071	41260	310.03	
Isoform 2 of amyloid-like protein 1	sp|P51693-2	713	72202	308.88	[6,9]
Ig gamma-1 chain C region	sp|P01857	1575	36083	292.89	
Fibulin-1	sp|P23142	1138	77162	281.56	[2]
Complement C2	sp|P06681	662	83214	265.89	[6,9]
Fibulin 2	tr|F5H1F3	77	129370	238.93	[10]
Complement factor H	sp|P08603	2007	139005	230.17	[11]
Ig lambda chain V-I region HA	sp|P01700	156	11889	182.16	
Neurexin-2-alpha	sp|Q9P2S2	62	184865	166.39	[3]
Complement C1s subcomponent	sp|P09871	1046	76635	163.60	
Ig lambda chain V-III region SH	sp|P01714	80	11386	160.25	
Complement component C6	sp|P13671	480	104718	151.22	[12]
Ig kappa chain C region	sp|P01834	1607	11602	109.59	
Ig gamma-2 chain C region	sp|P01859	1131	35878	90.07	

Isoform 15 of Fibronectin	sp|P02751-15	8651	272150	89.01	
Hornerin	sp|Q86YZ3	40	282228	82.95	
NELL2	tr|F8VVB6	1143	85863	77.62	[13]
Ig lambda chain V-IV region Bau	sp|P01715	37	11298	75.91	
Complement C3	sp|P01024	6034	187030	75.35	[3]
ROBO1 protein	tr|B2RXI1	373	175727	58.46	[14]
Angiotensinogen	sp|P01019	356	53121	50.00	[11]
Brevican (Fragment)	tr|Q5T3I6	59	24898	42.99	[15]
Contactin-associated protein-like 4	tr|F5H107	109	139833	40.66	
Ig kappa chain V-III region SIE	sp|P01620	423	11768	39.88	
Collagen alpha-1(I) chain	sp|P02452	296	138857	36.11	[3]
Ig kappa chain V-III region VG (Fragment)	sp|P04433	68	12567	34.66	
Serpin peptidase inhibitor, clade G (C1 inhibitor), member 1	tr|B4E1F0	1302	55734	32.35	
Fibrinogen γ chain, isoform γ-A	sp|P02679-2	820	49465	31.76	[11]
Ig heavy chain V-III region BRO	sp|P01766	738	13218	30.96	
Uncharacterized protein	tr|C9IZD4	180	141319	30.03	
Ig kappa chain V-I region AG	sp|P01593	182	11985	29.66	
NEO1 protein	tr|B7ZKM9	755	154207	28.96	
Contactin-1, isoform 2	sp|Q12860-2	1846	111796	27.81	[3]
CSPG3 variant protein (Fragment)	tr|Q4LE67	1200	144552	26.99	
Isoform 2 of Sulfhydryl oxidase 1	sp|O00391-2	36	66818	26.00	
Collagen, type III, alpha 1	tr|E7ENY8	85	111928	24.15	
IgGFc-binding protein	sp|Q9Y6R7	209	571639	24.11	
Ig heavy chain V-III region TUR	sp|P01779	593	12423	23.27	
Beta-2-glycoprotein 1	sp|P02749	54	38273	23.06	[16]
Collagen, type I, alpha 2	tr|F5H299	116	129445	22.22	[9]

Alpha-1-antitrypsin	sp|P01009	1340	46707	21.44	[11]
Prothrombin	sp|P00734	122	69992	21.03	[9]
Amyloid beta (A4) precursor protein	tr|B4DII8	918	84468	20.11	
Complement C4-B	sp|P0C0L5	2358	192673	18.65	
Complement component C4B (Childo blood group)	tr|A2BHY4	2445	192677	18.52	
Neurexin-3-alpha	sp|Q9Y4C0	465	180484	16.19	
Isoform 2 of Transmembrane protein 132A	sp|Q24JP5-2	524	110128	16.19	
Neural cell adhesion molecule L1-like protein	sp|O00533	1545	134987	16.11	
Netrin receptor DCC	sp|P43146	445	158357	15.60	
Neurofascin	tr|F8W791	370	148313	15.39	
Contactin-2	sp|Q02246	531	113322	15.27	
Vasorin	sp|Q6EMK4	176	71668	14.69	
Histidine-rich glycoprotein	sp|P04196	227	59541	13.54	
Seizure related 6 homolog (Mouse)-like	tr|B0QYH4	304	79590	13.50	
Isoform 2 of Nidogen-1	sp|P14543-2	154	121941	12.80	
Collagen alpha-1(VI) chain	sp|P12109	128	108462	12.69	
Ceruloplasmin	sp|P00450	2332	122128	12.68	
L1 cell adhesion molecule, isoform CRA_a	tr|G3XAF4	135	138821	12.33	
Inter-alpha (Globulin) inhibitor H2	tr|Q5T985	624	105150	11.75	
Ig alpha-1 chain C region	sp|P01876	630	37631	10.88	
Mannosidase, alpha, class 2A, member 2	tr|H0YKU9	79	120701	10.86	
Neural cell adhesion molecule 2	sp|O15394	782	92988	10.59	
Apolipoprotein A-I	sp|P02647	152	30759	10.09	
Phospholipid transfer protein	tr|B3KUE5	59	56598	9.10	
Uncharacterized protein	tr|F5H7E1	746	72076	8.95	
Isoform 2 of Ig mu chain C region	sp|P01871-2	127	51758	8.93	

Cystatin-C	sp|P01034	164	15789	8.53
Cartilage oligomeric matrix protein	tr|G3XAP6	21	79646	8.45
V-abl Abelson murine leukemia viral oncogene homolog 2	tr|D1MPS6	22	124482	8.14
Serpin peptidase inhibitor, clade A (Alpha-1 antiproteinase, antitrypsin), member 3, isoform CRA_b	tr|G3V5I3	377	50566	7.98
Prostaglandin D2 synthase 21kDa (brain) (Fragment)	tr|H0Y5A1	679	22932	7.49
Cell surface glycoprotein MUC18	sp|P43121	118	71563	7.41
Isoform 2 of Neural cell adhesion molecule 1	sp|P13591-1	1240	93303	7.09
Pigment epithelium-derived factor	sp|P36955	276	46283	6.84
Seizure-related 6 homolog (mouse) (Fragment)	tr|F5GZF9	438	98380	6.64
Protein Shroom3	sp|Q8TF72	64	216724	5.03
Protease, serine, 1 (trypsin 1)	tr|E7EQ64	66	28105	4.82
Kininogen 1	tr|B4DPP8	35	46467	4.75
Calcium channel, voltage-dependent, alpha 2/delta subunit 1	tr|F8WC36	1117	125311	4.47
Neuronal cell adhesion molecule	tr|E9PDA4	1027	131662	3.79
Apolipoprotein E	sp|P02649	525	36132	3.49
Laminin subunit gamma-1	sp|P11047	180	177489	2.95
Vitronectin	sp|P04004	34	54271	2.63
Hemopexin	sp|P02790	102	51643	2.23
CD163 molecule	tr|F5GZZ9	206	120244	2.21
Lumican	sp|P51884	62	38405	2.02
Serotransferrin	sp|P02787	4179	77014	1.22
Plexin domain-containing 2	tr|F5H554	63	58147	1.06
Delta and Notch-like epidermal growth factor-related receptor	sp|Q8NFT8	95	78422	0.59
Serum albumin	sp|P02768	8436	69321	0.57

SPARC-like 1 (hevin)	tr|B4E2Z0	233	61722	0.57
Seizure-related 6 homolog (mouse)-like 2	tr|F5H293	425	94410	0.50
Afamin	sp|P43652	223	69024	0.26
Dermcidin isoform 2	tr|A5JHP3	257	12406	0.20
Isoform 2 of Peptidase inhibitor 16	sp|Q6UXB8-2	75	29654	0.07

Accession, accession number in SwissProt/Tremble data base; Score, Mascot Score; R, mass spectrometry signal intensity ratio $I_{column-bound}/I_{depleted}$; Reference, reference suggesting eligibility as a biomarker

Reference List

1. Cooksley-Decasper S, Reiser H, Thommen DS, Biedermann B, Neidhart M, Gawinecka J *et al*. Antibody Phage Display Assisted Identification of Junction Plakoglobin as a Potential Biomarker for Atherosclerosis**.** PLoS ONE. 2012;7:e47985.

2. Hasselblatt M, Bohm C, Tatenhorst L, Dinh V, Newrzella D, Keyvani K *et al*. Identification of novel diagnostic markers for choroid plexus tumors - A microarray-based approach**.** Am J Surg Pathol. 2006;30:66-74.

3. Noben JP, Dumont D, Kwasnikowska N, Verhaert P, Somers V, Hupperts R *et al*. Lumbar cerebrospinal fluid proteome in multiple sclerosis: Characterization by ultrafiltration, liquid chromatography, and mass spectrometry**.** J Proteome Res. 2006;5:1647-57.

4. Tanaka M, Kikuchi H, Ishizu T, Minohara M, Osoegawa M, Motornura K *et al*. Intrathecal upregulation of granulocyte colony stimulating factor and its neuroprotective actions on motor neurons in amyotrophic lateral sclerosis**.** J Neuropath Exp Neur. 2006;65:816-25.

5. Blum-Degen D, Muller T, Kuhn W, Gerlach M, Przuntek H, Riederer P. Interleukin-1 beta and interleukin-6 are elevated in the cerebrospinal fluid of Alzheimer's and de novo Parkinson's disease patients**.** Neurosci Lett. 1995;202:17-20.

6. Kroksveen AC, Opsahl JA, Aye TT, Ulvik RJ, Berven FS. Proteomics of human cerebrospinal fluid: Discovery and verification of biomarker candidates in neurodegenerative diseases using quantitative proteomics**.** J Proteomics. 2011;74:371-88.

7. Hammack BN, Fung KYC, Hunsucker SW, Duncan MW, Burgoon MP, Owens GP *et al*. Proteomic analysis of multiple sclerosis cerebrospinal fluid**.** Mult Scler. 2004;10:245-60.

8. Yoshioka T, Kurokawa M, Sato T, Nagai K, Iizuka N, Arito M *et al*. Protein profiles of peripheral blood mononuclear cells as a candidate biomarker for Behcet's disease**.** Clin Exp Rheumatol. 2014;32:S9-S19.

9. Abdi F, Quinn JF, Jankovic J, McIntosh M, Leverenz JB, Peskind E *et al*. Detection of biomarkers with a multiplex quantitative proteomic platform in cerebrospinal fluid of patients with neurodegenerative disorders**.** J Alzheimers Dis. 2006;9:293-348.

10. Whiteaker JR, Zhang H, Zhao L, Wang P, Kelly-Spratt KS, Ivey RG *et al*. Integrated pipeline for mass spectrometry-based discovery and confirmation of biomarkers demonstrated in a mouse mode of breast cancer**.** J Proteome Res. 2007;6:3962-75.

11. Yin GN, Lee HW, Cho JY, Suk K. Neuronal pentraxin receptor in cerebrospinal fluid as a potential biomarker for neurodegenerative diseases**.** Brain Res. 2009;1265:158-70.

12. Schrag M, McAuley G, Pomakian J, Jiffry A, Tung S, Mueller C *et al*. Correlation of hypointensities in susceptibility-weighted images to tissue histology in dementia patients with cerebral amyloid angiopathy: a postmortem MRI study**.** Acta Neuropathol. 2010;119:291-302.

13. Roy S, Josephson SA, Fridlyand J, Karch J, Kadoch C, Karrim J *et al*. Protein biomarker identification in the CSF of patients with CNS lymphoma**.** J Clin Oncol. 2008;26:96-105.

14. Parray A, Siddique HR, Kuriger JK, Mishra SK, Rhim JS, Nelson HH *et al*. ROBO1, a tumor suppressor and critical molecular barrier for localized tumor cells to acquire invasive phenotype: Study in African-American and Caucasian prostate cancer models**.** Int J Cancer. 2014;135:2493-506.

15. Comabella M, Fernandez M, Martin R, Rivera-Vallve S, Borras E, Chiva C *et al*. Cerebrospinal fluid chitinase 3-like 1 levels are associated with conversion to multiple sclerosis**.** Brain. 2010;133:1082-93.

16. Gao Y, Wu K, Xu Y, Zhou H, He W, Zhang W *et al*. Characterization of Acute Renal Allograft Rejection by Human Serum Proteomic Analysis**.** J Huazhong Univ Sci Technolog Med Sci. 2009;29:585-91.
