# Supplementary material for: Depletion of highly abundant proteins from human cerebrospinal fluid: a cautionary note
Source: Mol Neurodegener. 2015 Oct 15;10:53. doi: 10.1186/s13024-015-0050-7 (PMC4608131; doi:10.1186/s13024-015-0050-7)
Supplement: Additional file 4: Table S2. — Proteins identified in fractions obtained by antibody/Protein G-based depletion (Accession, accession number in SwissProt/Tremble data base; R, MS signal intensity ratio Icolumn-bound/Idepleted; Score, Mascot Score; Reference, reference suggesting eligibility as a biomarker). (PDF 27 kb) [file 13024_2015_50_MOESM4_ESM.pdf]

Title: Depletion of highly abundant proteins from human cerebrospinal fluid: a cautionary note

Journal: Molecular Neurodegeneration

Authors: Ramona Günther, Eberhard Krause, Michael Schümann, Ingolf E. Blasig, Reiner F. Haseloff

Corresponding author: Reiner F. Haseloff, Leibniz Institute of Molecular Pharmacology, Robert-Roessle-Str. 10, D-13125 Berlin, Germany; email, haseloff@fmp-berlin.de

Supplementary Table 2: Proteins co-depleted in eluate/flow-through fractions obtained by antibody/protein G-based albumin/Ig depletion of human cerebrospinal fluid

**Protein**	**Accession**	**Score**	**Mass (Da)**	**R**	**Reference**
Coagulation factor XII	sp|P00748	40	67748	124.37	[1]
Desmoglein 1	sp|Q02413	393	113676	78.16	[2]
Lactotransferrin	tr|B7Z4X2	110	73114	36.96	[3]
Calmodulin-like protein 5	sp|Q9NZT1	93	15883	29.72	[2]
Collagen alpha-1(I) chain	sp|P02452	377	138857	9.57	[2]
Protein Shroom3	sp|Q8TF72	63	216724	4.26	[4]
Collagen, type I, alpha 2	tr|F5H299	398	129445	3.93	[5]

Protease, serine, 1 (trypsin 1)	tr|E7EQ64	70	28105	3.53	
Uncharacterized protein	tr|F8WES6	179	13100	2.65	
ATPase family AAA domain-containing protein 3C	sp|Q5T2N8	45	46350	2.37	
Ig kappa chain C region	sp|P01834	628	11602	1.07	
Ig gamma-1 chain C region	sp|P01857	1738	36083	1.02	
Ig gamma-2 chain C region	sp|P01859	1381	35878	0.98	
Ig gamma-3 chain C region	sp|P01860	1143	41260	0.93	
Complement factor H	sp|P08603	1959	139005	0.81	[6]
Ig kappa chain V-III region SIE	sp|P01620	249	11768	0.76	
Plasminogen	sp|P00747	1055	90510	0.74	[2]
Immunoglobulin lambda-like polypeptide 5	sp|B9A064	189	23049	0.68	
Ig heavy chain V-III region BRO	sp|P01766	627	13218	0.67	
Ig lambda-2 chain C regions	sp|P0CG05	283	11287	0.62	
Alpha-1-antitrypsin	sp|P01009	2469	46707	0.61	[6]
Ig kappa chain V-IV region Len	sp|P01625	99	12632	0.55	
Isoform 2 of Calsyntenin-1	sp|O94985-2	1752	108574	0.54	[6]
Serum albumin	sp|P02768	6983	69321	0.51	
Isoform 2 of Nidogen-1	sp|P14543-2	301	121941	0.51	
Complement component C6	sp|P13671	644	104718	0.43	

Isoform 2 of Reelin	sp|P78509-2	518	387951	0.38
Apolipoprotein A-I	sp|P02647	285	30759	0.29
Isoform 2 of Cartilage acidic protein 1	sp|Q9NQ79-2	79	70389	0.24
Complement factor B	tr|B4E1Z4	2052	140853	0.24
Cystatin-C	sp|P01034	95	15789	0.23
Serpin peptidase inhibitor, clade A, member 3, isoform CRA_b	tr|G3V5I3	2043	50566	0.23
CSPG3 variant protein (Fragment)	tr|Q4LE67	886	144552	0.23
Isoform 2 of Ig mu chain C region	sp|P01871-2	204	51758	0.23
Complement C2	sp|P06681	457	83214	0.23
Inter-alpha (Globulin) inhibitor H2	tr|Q5T985	937	105150	0.21
Gelsolin	sp|P06396	2274	85644	0.21
Prothrombin	sp|P00734	156	69992	0.21
Neurexin 1	tr|F5GYC7	1100	164607	0.21
Uncharacterized protein	tr|F5H7E1	571	72076	0.20
Vitronectin	sp|P04004	29	54271	0.19
Prostaglandin D2 synthase 21kDa (brain) (Fragment)	tr|H0Y5A1	1040	22932	0.18
Fibulin-1	sp|P23142	2116	77162	0.18
Ig alpha-1 chain C region	sp|P01876	1127	37631	0.17

Protein tyrosine phosphatase, receptor type, S	tr|H0YG31	175	174506	0.17
Pigment epithelium-derived factor	sp|P36955	900	46283	0.17
Complement component C4B (Childo blood group)	tr|A2BHY4	2818	192677	0.16
Ceruloplasmin	sp|P00450	5870	122128	0.16
Complement C3	sp|P01024	6867	187030	0.16
Coagulation factor V	sp|P12259	477	251546	0.15
Complement C1r subcomponent	sp|P00736	748	80067	0.15
Isoform 2 of Sulfhydryl oxidase 1	sp|O00391-2	222	66818	0.15
NEO1 protein	tr|B7ZKM9	1205	154207	0.14
Collagen alpha-1(VI) chain	sp|P12109	426	108462	0.14
Ectonucleotide pyrophosphatase/phosphodiesterase 2	tr|E7EUF1	1167	101488	0.14
EGF containing fibulin-like extracellular matrix protein 1	tr|B4DW75	57	39169	0.14
Semaphorin-7A	sp|O75326	273	74776	0.13
Kallikrein B, plasma (Fletcher factor) 1 (Fragment)	tr|H0YAC1	189	76834	0.13
Lumican	sp|P51884	323	38405	0.12
Complement component 4A (Rodgers blood group)	tr|B0UZ83	2792	192754	0.12
L1 cell adhesion molecule, isoform CRA_a	tr|G3XAF4	285	138821	0.12
Vitamin K-dependent protein S	sp|P07225	323	75074	0.11
Serpin peptidase inhibitor, clade G (C1 inhibitor), member 1	tr|B4E1F0	2208	55734	0.11

Apolipoprotein E	sp|P02649	1340	36132	0.10
Isoform 2 of Transmembrane protein 132A	sp|Q24JP5-2	446	110128	0.10
Contactin-2	sp|Q02246	1619	113322	0.10
Hemopexin	sp|P02790	894	51643	0.09
Alpha-2-macroglobulin	tr|H0YGH4	10961	164837	0.09
Hornerin	sp|Q86YZ3	27	282228	0.09
IgGFc-binding protein	sp|Q9Y6R7	548	571639	0.09
Calcium channel, voltage-dependent, alpha 2/delta subunit 1	tr|F8WC36	2846	125311	0.09
Isoform 2 of Neural cell adhesion molecule 1	sp|P13591-1	3584	93303	0.08
Kinesin-like protein KIF14	sp|Q15058	20	186375	0.08
Plexin domain-containing 2	tr|F5H554	147	58147	0.08
Phospholipid transfer protein	tr|B3KUE5	344	56598	0.08
Neural cell adhesion molecule 2	sp|O15394	1002	92988	0.07
Alpha-1B-glycoprotein	sp|P04217	483	54220	0.07
Complement component C7	sp|P10643	1201	93457	0.07
Neuronal cell adhesion molecule	tr|E9PDA4	3131	131662	0.07
Activated leukocyte cell adhesion molecule	tr|F5GXJ9	138	59491	0.06
Contactin 6	tr|F5H752	329	105771	0.06
Cell surface glycoprotein MUC18	sp|P43121	505	71563	0.06

Neurofascin	tr|F8W791	940	148313	0.06
Contactin-3	sp|Q9P232	285	112812	0.06
Inter-alpha-trypsin inhibitor heavy chain family, member 4	tr|E9PGN5	2350	99795	0.05
Histidine-rich glycoprotein	sp|P04196	653	59541	0.05
Group-specific component (vitamin D-binding protein)	tr|D6RAK8	256	55041	0.05
Isoform 2 of Amyloid-like protein 1	sp|P51693-2	1269	72202	0.05
Serotransferrin	sp|P02787	8978	77014	0.04
Delta and Notch-like epidermal growth factor-related receptor	sp|Q8NFT8	142	78422	0.04
Neural cell adhesion molecule L1-like protein	sp|O00533	2769	134987	0.04
Seizure-related 6 homolog (mouse)-like 2	tr|F5H293	803	94410	0.04
Vascular cell adhesion molecule 1	tr|E9PDD1	492	74299	0.04
Uncharacterized protein	tr|C9IZD4	664	141319	0.03
Kininogen 1	tr|B4DPP8	463	46467	0.03
Cadherin 6, type 2, K-cadherin (fetal kidney)	tr|D6RF86	226	67361	0.03
Amyloid-like protein 1	sp|P51693	1241	72131	0.03
CD163 molecule	tr|F5GZZ9	672	120244	0.02
Peptidase inhibitor 16	sp|Q6UXB8	167	49440	0.02
Afamin	sp|P43652	589	69024	0.02

Isoform 4 of Extracellular matrix protein 1	sp|Q16610-4	150	63523	0.01
Dickkopf 3 homolog (Xenopus laevis)	tr|F6SYF8	651	39924	0.01
Cadherin 13, H-cadherin (heart)	tr|C9JRI6	297	78253	0.01

Accession, accession number in SwissProt/Tremble data base; Score, Mascot Score; R, mass spectrometry signal intensity ratio $I_{column\text{-}bound}/I_{depleted}$; Reference, reference suggesting eligibility as a biomarker

Reference List

1. Kraft P, De Meyer S.F., Kleinschnitz C. Next-generation antithrombotics in ischemic stroke: preclinical perspective on 'bleeding-free antithrombosis'**.** J Cereb Blood Flow Metab. 2012;32:1831-40.

2. Noben JP, Dumont D, Kwasnikowska N, Verhaert P, Somers V, Hupperts R *et al*. Lumbar cerebrospinal fluid proteome in multiple sclerosis: Characterization by ultrafiltration, liquid chromatography, and mass spectrometry**.** J Proteome Res. 2006;5:1647-57.

3. Fillebeen C, Ruchoux MM, Mitchell V, Vincent S, Benaissa M, Pierce A. Lactoferrin is synthesized by activated microglia in the human substantia nigra and its synthesis by the human microglial CHME cell line is upregulated by tumor necrosis factor alpha or 1-methyl-4-phenylpyridinium treatment**.** Mol Brain Res. 2001;96:103-13.

4. Aarhus M, Helland C.A., Lund-Johansen M, Wester K, Knappskog PM. Microarray-based gene expression profiling and DNA copy number variation analysis of temporal fossa arachnoid cysts**.** Cerebrospinal Fluid Res. 2010;7:6.

5. Abdi F, Quinn JF, Jankovic J, McIntosh M, Leverenz JB, Peskind E *et al*. Detection of biomarkers with a multiplex quantitative proteomic platform in cerebrospinal fluid of patients with neurodegenerative disorders**.** J Alzheimers Dis. 2006;9:293-348.

6. Yin GN, Lee HW, Cho JY, Suk K. Neuronal pentraxin receptor in cerebrospinal fluid as a potential biomarker for neurodegenerative diseases**.** Brain Res. 2009;1265:158-70.
